# High seroprevalence of SARS-CoV-2 in Burkina-Faso, Ghana and Madagascar in 2021: a population-based study

**DOI:** 10.1186/s12889-022-13918-y

**Published:** 2022-09-05

**Authors:** Nicole S. Struck, Eva Lorenz, Christina Deschermeier, Daniel Eibach, Jenny Kettenbeil, Wibke Loag, Steven A. Brieger, Anna M. Ginsbach, Christian Obirikorang, Oumou Maiga-Ascofare, Yaw Adu Sarkodie, Eric Ebenezer Amprofi Boham, Evans Asamoah Adu, Gracelyn Asare, Amos Amoako-Adusei, Alfred Yawson, Alexander Owusu Boakye, James Deke, Nana Safi Almoustapha, Louis Adu-Amoah, Ibrahim Kwaku Duah, Thierry A. Ouedraogo, Valentin Boudo, Ben Rushton, Christa Ehmen, Daniela Fusco, Leonard Gunga, Dominik Benke, Yannick Höppner, Zaraniaina Tahiry Rasolojaona, Tahinamandranto Rasamoelina, Rivo A. Rakotoarivelo, Raphael Rakotozandrindrainy, Boubacar Coulibaly, Ali Sié, Anthony Afum-Adjei Awuah, John H. Amuasi, Aurélia Souares, Jürgen May

**Affiliations:** 1 Infectious Disease Epidemiology, Bernhard Nocht Insitute for Tropical Medicine, Hamburg, Germany; 2 German Center for Infection Research (DZIF), Hamburg-Borstel-Lübeck-Riems, Heidelberg, Germany; 3 Institute of Medical Biostatistics, Epidemiology and Informatics, University Medical Centre of the Johannes Gutenberg, University Mainz, Mainz, Germany; 4 Diagnostics Development Laboratory, Bernhard Nocht Institute for Tropical Medicine, Hamburg, Germany; 5 University of Sussex Business School, University of Sussex, Falmer, UK; 6 Kumasi Centre for Collaborative Research in Tropical Medicine, Kwame Nkrumah University of Science and Technology, Kumasi, Ghana; 7 Department of Molecular Medicine, Kwame Nkrumah University of Science and Technology, Kumasi, Ghana; 8 Department of Clinical Microbiology, Kwame Nkrumah University of Science and Technology, Kumasi, Ghana; 9 Department of Community Health, University of Ghana, Accra, Ghana; 10 Centre de Recherche en Santé de Nouna, Nouna, Burkina Faso; 11 Centre d’Infectiologie Charles Méreiux, Antananarivo, Madagascar; 12 University of Fianarantsoa, Fianarantsoa, Madagascar; 13 University of Antananarivo, Antananarivo, Madagascar; 14 Heidelberg Institute of Global Health (HIGH), Heidelberg University Hospital, Heidelberg University, Heidelberg, Germany; 15 Department of Global and International Health, Kwame Nkrumah University of Science and Technology, Kumasi, Ghana; 16 Department of Tropical Medicine I, University Medical Center Hamburg-Eppendorf (UKE), Hamburg, Germany

**Keywords:** SARS-CoV-2, Seroprevalence, Population-based, Sub-Saharan Africa, Bayesian model

## Abstract

**Background:**

The current COVID-19 pandemic affects the entire world population and has serious health, economic and social consequences. Assessing the prevalence of COVID-19 through population-based serological surveys is essential to monitor the progression of the epidemic, especially in African countries where the extent of SARS-CoV-2 spread remains unclear.

**Methods:**

A two-stage cluster population-based SARS-CoV-2 seroprevalence survey was conducted in Bobo-Dioulasso and in Ouagadougou, Burkina Faso, Fianarantsoa, Madagascar and Kumasi, Ghana between February and June 2021. IgG seropositivity was determined in 2,163 households with a specificity improved SARS-CoV-2 Enzyme-linked Immunosorbent Assay. Population seroprevalence was evaluated using a Bayesian logistic regression model that accounted for test performance and age, sex and neighbourhood of the participants.

**Results:**

Seroprevalence adjusted for test performance and population characteristics were 55.7% [95% Credible Interval (CrI) 49·0; 62·8] in Bobo-Dioulasso, 37·4% [95% CrI 31·3; 43·5] in Ouagadougou, 41·5% [95% CrI 36·5; 47·2] in Fianarantsoa, and 41·2% [95% CrI 34·5; 49·0] in Kumasi. Within the study population, less than 6% of participants performed a test for acute SARS-CoV-2 infection since the onset of the pandemic.

**Conclusions:**

High exposure to SARS-CoV-2 was found in the surveyed regions albeit below the herd immunity threshold and with a low rate of previous testing for acute infections. Despite the high seroprevalence in our study population, the duration of protection from naturally acquired immunity remains unclear and new virus variants continue to emerge. This highlights the importance of vaccine deployment and continued preventive measures to protect the population at risk.

**Supplementary Information:**

The online version contains supplementary material available at 10.1186/s12889-022-13918-y.

## Background

As at February 7^th^, 2022 the COVID-19 pandemic has resulted in more than 390 million cases and 5.7 million deaths worldwide [1]. Official counts of COVID-19 cases and deaths have suggested a moderate morbidity in sub-Saharan Africa. Burkina Faso, Madagascar and Ghana reported their first cases on March 9^th^, March 20^th^ and March 12^th^, 2020, respectively, and almost two years later have reported 20,729, 62,844 and 158,159 confirmed cases [https://covid19.who.int/, accessed on February 17^th^ 2022]. Mild and asymptomatic infections are often not readily captured in passive surveillance activities, which are used to track and report on disease epidemiology in resource-constrained settings. Therefore, it is likely that reported cases underestimate progression of the disease in these settings. As such, population-based serological surveys that measure antibodies against SARS-CoV-2 can determine the proportion of the population that has been exposed to the virus, with or without symptoms [2]. This information is valuable in order to evaluate control and prevention measures and inform health policy decisions. At the time of writing, a global SARS-CoV-2 seroprevalence tracker [3] recorded 2,812 serosurveys worldwide. Of those, 242 had been conducted in 28 African countries. The majority of peer reviewed seroprevalence studies in Africa targeted specific sub-populations, occupational sectors or tested blood donors. Although such convenience-based sampling strategies allow for rapid answers in uncertain pandemic-driven times, they cannot provide accurate seroprevalence estimates for a general population. To date, there are only 35 studies in 17 African countries that applied cross-sectional community-based sampling strategies. The majority used lateral flow immunoassays (LFIA) to assess SARS-CoV-2 seropositivity, whereas only five used Enzyme-linked immunosorbent assays (ELISA). The former method is low-cost, simple and fast but prone to false-negative or false-positive results [4]. The latter is comparably more complex in its procedure, longer in detection time and more expensive but ensures higher sensitivity and specificity [5]. It has been shown, however, that some commercial ELISAs are prone to low specificity in malaria-endemic regions [6]. Conducting seroprevalence surveys with robust methods in terms of representativeness of the target population and the use of validated tests that are highly sensitive and specific is important. The primary objective of our study was to estimate the proportion of the sampled population that had been exposed to the virus. We measured IgG seropositivity against SARS-CoV-2 based on specificity-improved ELISA assays [7] from blood plasma and adjusted estimates for test performance and population characteristics, in order to determine the progression of the epidemic and an improved measure of seroprevalence in the target cities.

## Methods

### Study design and participants

The SeroCoV study protocol has been published [8]. The study was conducted in four study sites, Bobo Dioulasso and Ouagadougou in Burkina Faso, Fianarantsoa in Madagascar and Kumasi in Ghana between February and June 2021. Study sites were selected in consultation with local investigators based on laboratory infrastructures, accessibility to communities and staff safety. Urban sites were selected over rural ones as more cases were registered in urban areas in the official systems. In summary, a household-based cross-sectional seroprevalence survey was performed in a population of individuals aged 10 years and older living in urban areas using a two-stage cluster geo-point sampling approach. In the first stage, administrative boundaries were used to allocate clusters, which were selected based on the probability proportional to population size (PPS) method. In the second stage, geographical coordinates were randomly selected within clusters. After navigation to the coordinates, sampling teams identified eligible households by following pre-defined standard operating procedures, taking different scenarios into account: A GPS coordinate falling on exactly one household, multiple households, or no household (SOP available in Appendix 1). Household members were eligible for inclusion if informed consent was obtained. A minimum age of 10 years and a requirement of no existing health problems contraindicating blood sample collection were applied. The sample size was calculated in relation to the primary outcome and based on one individual per household, yielding an actual sample size of 557 per site. To account for 15% non-response of eligible households, we adjusted the number of recruited households to 557/0.85 = 655. One person per household was included in the study in line with the countries' age and gender distribution. Ethical clearance was obtained by the National Ethical Board Committees of each participating country. Informed consent was obtained from all individuals before data and sample collection. The risks and side effects from blood drawing were explained, and participants were given the right to withdraw from the study at any time. No information on the vaccination status of the study participants was collected at the time of recruitment as the roll out of the vaccination campaigns only began after completion of recruitment and sample collection. The mass vaccination of the population in Ghana started in May 2021 [9], in July 2021 in Madagascar [10], and Burkina Faso only received vaccine doses in November 2021 [11].

### Laboratory analyses and ELISA testing

Enzyme-linked Immunosorbent Assays (ELISAs) were performed on plasma samples using an ELISA assay based on a patented platform technology [12] (Patent EP2492689) developed at the Bernhard Nocht Institute for Tropical Medicine, Hamburg, Germany [7]. As antigen, a recombinantly expressed, truncated SARS-CoV-2 nucleocapsid protein (NCP) was employed. Assay specificity was determined using 790 pre-COVID-19 serum samples originating from Europe, Africa, South America, and Asia during assay validation and reassessed in the present study using locally acquired pre-COVID-19 stock samples from Burkina Faso (*N* = 93) and Ghana (*N*= 536). Assay sensitivity was determined during ELISA validation using longitudinal serum samples obtained between day 10 and day 446 post-onset of symptoms from German patients with a PCR-confirmed SARS-CoV-2 infection. No cross-reactivity with antibodies elicited by previous infections with common cold Coronaviruses was observed [7].

### Statistical analyses

The primary outcome measure was the proportion of the study population that has been exposed to the virus measured as IgG seropositivity against SARS-CoV-2 based on ELISAs. Crude prevalence was calculated, as percentages of individuals with a positive test result in relation to the total number of individuals (result 'positive' compared to 'negative', 'undetermined'), with exact 95% Confidence Intervals (CI). To estimate seroprevalence along with 95% Credible Intervals (CrI), we used a Bayesian logistic regression model with post-stratification on age and sex of the population and administrative areas within each study region. Similar to confidence intervals (CI) in frequentist statistics, CrIs indicate the interval in which the unobserved parameter value falls with a particular certainty. The model therefore accounts for uncertainty around the specificity and sensitivity of the test in the priors and in the estimation of prevalence [13]. Prior information on test performance by the test developer was included by adding a hierarchical structure to the model of both specificity and sensitivity. Details are provided in the statistical appendix [Appendices 2 and 3].

For categorical variables, numbers and percentages are presented for each of the four study sites [14]. Continuous variables were described using median and interquartile range (IQR). Missing age information was imputed by random draws of the approximate age distribution (right-skewed log normal distribution) in the remaining participants.

## Results

### Study population

Altogether 2,540 households were visited between February 2^nd^ and June 18^th^ 2021 in Bobo-Dioulasso, Ouagadougou, Fianarantsoa, and Kumasi (Table 1). Of those, 2,434 consented to participate and 2,163 were eligible for analyses with one participant from each of the 627 households in Bobo-Dioulasso, 522 households in Ouagadougou, 674 households in Fianarantsoa, and 340 households in Kumasi, in line with the countries' age and gender distribution. Households were excluded from analysis in the case of ID mismatches. The median age [interquartile range, IQR] in the recruited study population was 26 [18–39], 32 [22–44], 30 [19–42], and 35 [23–51] in Bobo-Dioulasso, Ouagadougou, Fianarantsoa, and Kumasi, respectively. Females were equally represented in Bobo-Dioulasso (50.7%) and Ouagadougou (52.9%), but less so in Fianarantsoa (45.7%) and Kumasi (40.3%).

**Table 1:** Demographic characteristics of the participants in the final sample of 2,163 study participants

	**Bobo-Dioulasso**	**Ouagadougou**	**Fianarantsoa**	**Kumasi**
**Recruitment period**	03.02.2021 – 11.03.2021	02.02.2021 – 13.03.2021	26.02.2021 – 18.06.2021	17.02.2021 – 10.05.2021
**Screened households**	650	655	697	538
**Informed consent obtained**	645	654	676	459
**Number of households considered for analyses**^**f1**^	627	522	674	340
**Household demographics**				
**Sex**				
% female	318 / 627 (50·7%)	276 / 522 (52·9%)	308 / 674 (45·7%)	137 / 340 (40·3%)
**Age (in years) /sex strata (n/N (%)) **^**f2**^				
10 – 19, male	101 / 627 (16·1%)	41 / 522 (7·9%)	97 / 674 (14·4%)	30 / 340 (8·8%)
10 – 19, female	107 / 627 (17·1%)	61 / 522 (11·7%)	83 / 674 (12·3%)	32 / 340 (9·4%)
20 – 44, male	153 / 627 (24·4%)	145 / 522 (27·8%)	187 / 674 (27·7%)	53 / 340 (15·6%)
20 – 44, female	159 / 627 (25·4%)	156 / 522 (29·9%)	156 / 674 (23·2%)	107 / 340 (31·5%)
≥ 45, male	55 / 627 (8·8%)	60 / 522 (11·5%)	82 / 674 (12·2%)	54 / 340 (15·9%)
≥ 45, female	52 / 627 (8·3%)	59 / 522 (11·3%)	69 / 674 (10·2%)	64 / 340 ( 18·8%)

*n* Nominator of individuals in each stratum, *N* Denominator of individuals in each stratum

^f1^Discrepancy between households that consented to participate and those who were considered for analyses is explained by ID mismatches

^f2^Information on age is missing for 2 participants in Bobo-Dioulasso and 4 individuals in Kumasi and was replaced by random draws of the approximate age distribution (right-skewed log normal distribution) in the remaining participants

### Crude serological SARS-CoV-2 status and previous testing for acute infections

**Fig. 1:**
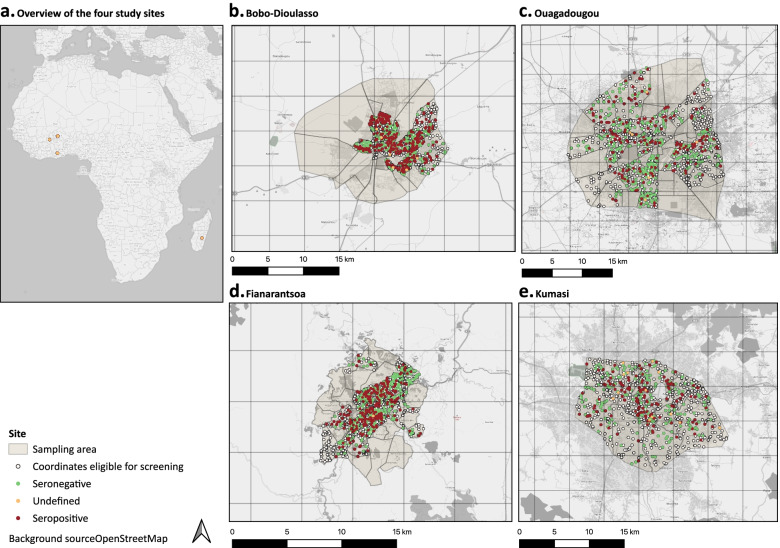
Overview of sampled area and serological status of study participants

Figure 1 shows a geographic overview of the four sampled cities and the distribution of coordinates from where eligible households were identified (Fig. 1, white circles). Seronegative and positive households are indicated as green and red circles, respectively.

The crude proportion of seropositive individuals at the time of recruitment and sampling was.

50·6% [95% CI 46·6; 54·5] in Bobo-Dioulasso, 32·6% [95% CI 28·6; 36·8] in Ouagadougou, 37·5% [95% CI 33·9; 41·3] in Fianarantsoa, and 38·8% [95% CI 33·6; 44·2] in Kumasi (Table 2). The number of undetermined results (i.e. measurements around the cut-off threshold) was below 1·2% in Burkina Faso and Madagascar, and 3·8% in Kumasi. The distribution of seropositive and seronegative individuals in the four sampled cities did not show obvious clustering (Fig. 1).

**Table 2:** Crude seropositivity and testing for acute infection

	**Bobo-Dioulasso**	**Ouagadougou**	**Fianarantsoa**	**Kumasi**
	n/N (%)	n/N (%)	n/N (%)	n/N (%)
**Crude seropositivity**	317 / 627 (50·6)^f1^	170 / 522 (32·6)^f2^	253 / 674 (37·5)^f3^	131 / 340 (38·8)^f4^
**No test for acute infection performed since onset of pandemic**	611 / 627 (97·4)	493 / 522 (94·4)	655 / 674 (97·2)	322 / 340 (94·7)
**Test for acute infection performed prior to survey**	16 / 627 (2·6)	29 / 522 (5·6)	19 / 674 (2·8)	18/340 (5·3)
**Positive results for acute infection among those tested**	2 / 16 (12·5)	1 / 29 (3·5)	4 / 19 (21·1)	2/18 (11·1)

*n* N, sample size

^f1^Of the remaining tested participants, 6 (1·0%) had an undefined and 304 (48·5%) a negative serological result

^f2^Of the remaining tested participants, 6 (1·2%) had an undefined and 346 (66·3%) a negative serological result

^f3^Of the remaining tested participants, 1 (0·2%) had an undefined and 420 (62·3%) a negative serological result

^f4^Of the remaining tested participants, 13 (3·8%) had an undefined and 196 (57·7%) a negative serological result

In Bobo-Dioulasso and Fianarantsoa, the frequency of seropositivity was higher in in the age groups 10–19 and 20–44 but not in the age group 45 and older (Supplemental Fig. 1 and Table 1). This was different in Ouagadougou, where the frequency of seropositivity was higher in females across the age groups 10–19 and 20–44 but lower in the oldest age group. In Kumasi, the seropositivity was higher in males in the age group 20–44 and 45 and older but lower in the age group 10–19. The frequency of seropositivity was consistently lower in the age group 20–44 in Ouagadougou and Fianarantsoa but similar across all age groups in Bobo-Dioulasso. In Kumasi, the frequency of seropositivity was similar between the age groups 10–19 and 20–44.

**Fig. 2:**
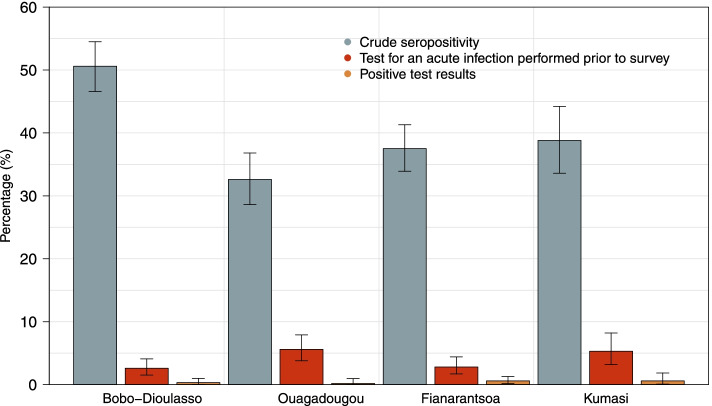
Crude seropositivity and tests performed to assess acute infection with respective result

The majority of study participants (97·4% in Bobo-Dioulasso, 94·4% in Ouagadougou, 97·2% in Fianarantsoa, and 94·7% in Kumasi) had reported to not have received a test for an acute SARS-CoV-2 infection prior to our survey (Table 2 and Fig. 2). Further, only a fraction (2/16 in Bobo-Dioulasso, 1/29 in Ouagadougou, 4/19 in Fianarantsoa and 2/18 in Kumasi) of those who were tested reported a positive result (Table 2 and Fig. 2).

### Adjusted SARS-CoV-2 seroprevalence

**Fig. 3:**
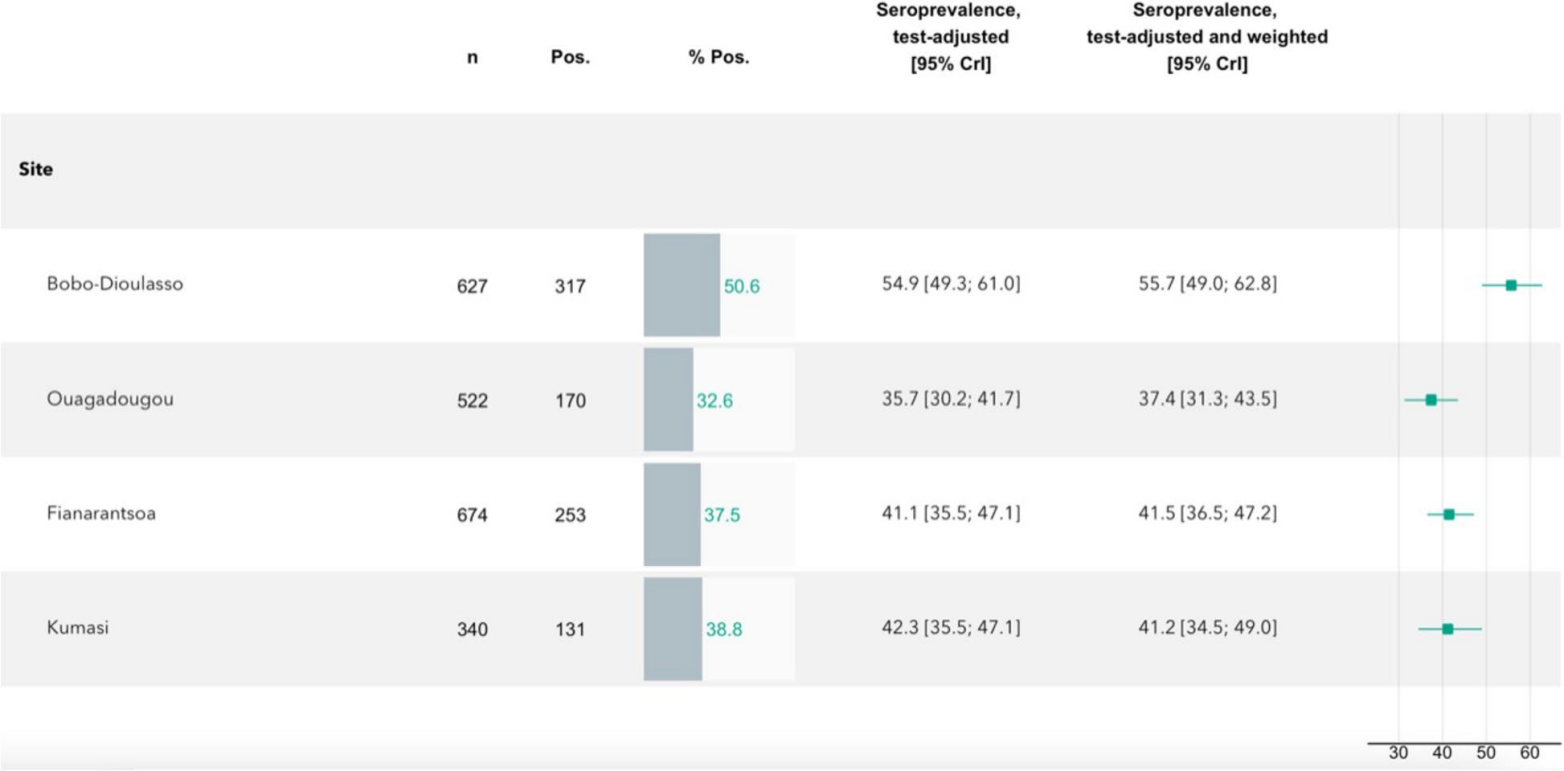
Estimated test-adjusted seroprevalence based on a Bayesian logistic regression model with post-stratification on age and sex of the population and administrative areas within each study region. Abbreviations: n, number of individuals in each site; Pos., positive; CrI, Credible Interval

After adjusting for sensitivity and specificity of the antibody test, the seroprevalence estimates increased across all cities to 54·9% [95% CrI 49·3; 61·0] in Bobo-Dioulasso, 35·7% [95% CrI 30·2 – 41·7] in Ouagadougou, 41·1% [95% CrI 35·5; 47·1] in Fianarantsoa, and 42·3% [95% CrI 35·5; 47·1] in Kumasi (Fig. 3). The post-stratification for age, sex, and administrative area then raised the overall seroprevalence estimates marginally, from 54·9 to 55·7% [95% CrI 49·0; 62.8] in Bobo-Dioulasso, from 35·7 to 37·4% [95% CrI 31·3; 43·5] in Ouagadougou, from 41·1 to 41·5% [95% CrI 35·5; 47·2] in Fianarantsoa, and decreased slightly from 42·3 to 41·2% [95% CrI 34·5; 49·0] in Kumasi.

## Discussion

In the present SARS-CoV-2 seroprevalence analysis, between 37 and 56% of the population in Bobo-Dioulasso, Ouagadougou, Fianarantsoa, and Kumasi had been exposed to the virus and exhibited antibodies against SARS-CoV-2 in the first half of 2021. Only a small proportion of the study population tested positive for an acute SARS-CoV-2 infection since the onset of the pandemic. Our findings point to an extensive but silent spread of SARS-CoV-2 in the three countries.

### Underestimation of acute infections

To date and since March 2020, two epidemic wave have been noted in Burkina Faso, three in Madagascar, and four in Ghana (https://ourworldindata.org/covid-cases [15], accessed on February 14^th^ 2021) [1]. The detection of epidemic waves relies on testing consistency [16]. Extrapolating the seropositivity rate of our study population onto the total population number in the targeted districts of Bobo-Dioulasso and Ouagadougou infers that 352,848 individuals in Bobo-Dioulasso and 579,162 individuals in Ouagadougou had been infected by March 2021. The combined seroprevalence estimate for these two cities is 75 times higher than the reported cumulated case count for the entire country at that time. These results correspond to findings from South Sudan [17] and Zambia [18], where the number of implied infections was found to be 100 and 92 times higher than the number of officially reported cases. Similarly, our extrapolated seroprevalence estimate for Fianarantsoa is twice as high as the official cumulated case number for the entire of Madagascar by June 2021, and nine times higher in Kumasi than the official cumulated case count for the entire of Ghana by May 2021. Reasons for the underestimation of infections may include insufficient testing capacities, limited access to testing, a lower acceptance of testing, and perhaps a low motivation of asymptomatic individuals to get tested, as there were no organized campaigns for large-scale testing [19]. Only individuals with symptoms suggestive of a SARS-CoV-2 infection, and people who travelled outside the country, were targeted for testing.

### Limited testing capacities

By October 2021, 4 billion COVID-19 tests for acute infection had been performed since the onset of the pandemic globally but only 1.8% were carried out in Africa [20]. According to the online tool developed by Ritchie and co-workers [15] on the SARS-CoV-2 tests performed worldwide, data for Burkina Faso has only become available since January 2022. Ghana’s testing rate showed a constant high turnover of tested samples since the onset of the pandemic and was among the highest in sub-Saharan Africa due to their applied testing approach of pooled samples [21]. Madagascar showed low but constant activity in tests performed until September 2021.

### Impact of SARS-CoV-2

To date, there are more than 5.8 million deaths due to COVID-19 worldwide (https://covid19.who.int/, accessed on February 15^th^2022) [1]. The three countries with the highest number of deaths are Italy (12,105,675 cases and 151,015 deaths), Spain (10,555,197 cases and 95,606 deaths), and the United States of America (76,983,188 cases and 910,982 deaths). In comparison, 7,942,093 cases and 162,673 deaths were recorded for the entire African continent. The perceived risk of contracting the disease might be lower in populations with a high proportion of asymptomatic cases. Therefore, safety measures, such as physical distancing and wearing masks may be even more difficult to enforce due to the low-risk perception of the population [22, 23]. Health policy makers would need to carefully consider how best to communicate the benefits of protective measures and vaccinations in settings where the majority of people have not experienced severe illness or even know anyone who has.

### Affected population groups

In Bobo-Dioulasso and Fianarantsoa, 10–19- and 20–45-year-old males were more exposed to the virus than females of the same age group, while females had higher SARS-CoV-2 seropositivity in the > 45-year-old group. This finding is consistent with previous studies documenting higher infection rates in young human males [24], which might be explained by differences in risk behaviors [25], and responsible attitudes toward the COVID-19 pandemic [26]. The higher level of SARS-CoV-2 seropositivity in older females might be related to having stronger ties to their family members, friends, co-workers, neighbours, and community [27]. However, the age and sex patterns for Ouagadougou and Kumasi were different.

### SARS-CoV-2 serosurveys

A recent systematic review and meta-analysis on SARS-CoV-2 serosurveys demonstrated that only 20% of the included 404 studies were of high quality based on a newly developed scoring system that included study design, laboratory assay, and outcome adjustment [28]. 64% of the study populations were convenience samples, and only 12% of the population-based studies achieved the highest score. At the time of writing, no population-based serological surveys for exposure to SARS-CoV-2 had been available for Burkina Faso, Madagascar, or Ghana. One study based on blood donations was available from Madagascar [29], and one study targeting different types of public locations and healthcare/research institutes was available from Ghana [30]. Both studies targeted specific sub-groups of the population so extrapolation of the prevalence to the urban community or general population is not valid.

### Policy implications

This study makes a strong case for the need of routine community-level seroprevalence studies as part of COVID-19 surveillance activities in order to inform vaccination schedules and details thereof, including planning for age, geographic location and socioeconomic groupings. Our findings further show that a more coordinated approach to vaccination strategies and SARS-COV-2 seroprevalence estimates across Africa is needed, as uneven vaccination rates (in particular with respect to low rates and waning seroprevalence) risk diluting the positive gains made by other countries which are able to achieve herd immunity thresholds via vaccination within reasonable time frames.

### Strengths of the study

This is the first study in Burkina Faso, Madagascar, and Ghana to assess the SARS-CoV-2 seroprevalence in a random sample of residents in urban settings. The age- and sex-stratified, two-stage cluster sampling approach increases the representativeness of the target population not only in terms of age and sex but possibly also for unmeasured confounders and minimizes the risk of selection bias. A highly sensitive and specific ELISA test, validated on local pre-pandemic serum samples, was used. Furthermore, a Bayesian hierarchical logistic regression model with post-stratification was applied for more accurate estimates of previous infections, and has reduced limitations linked to the sampling.

### Limitations of the study

A 15% refusal rate to participate was seen in Kumasi, which may have induced a selection bias. Further, underestimation of seroprevalence could be caused by post-infection antibody waning. Both Madagascar and Ghana experienced the first wave of SARS-CoV-2 infections between July and August 2020, while sampling was performed six to eleven months later. Antibodies against the nucleoprotein have been observed to drop significantly within 12 months post infection [31], so this may be another source for an underestimation.

## Conclusions

Overall, the study data suggest a much higher SARS-CoV-2 seroprevalence than anticipated through the reported national case numbers in the three countries. This information demonstrates that more accurate and timely information through active surveillance is necessary. There is a need to strengthen diagnostic facilities and to make them accessible for the general population, including sequencing capacities to monitor emerging variants. Nevertheless, the study population in neither of the cities was close to reaching the herd immunity threshold by the time of sampling and there is a lack of data from rural areas. The data show different age and sex distributions of seropositive individuals in the different study sites. This not only emphasizes the need for vaccinations for all age groups in the region, but the need for vaccination programs to be planned based on the prevailing seroprevalence distribution in order to maximize efficiency and achieve the best outcomes. This data-driven implementation strategy of SARS-CoV-2 vaccination programs is especially important in the light of the relatively short shelf-life of the vaccines. Vaccine nationalism and unequal vaccine distribution will exacerbate inequality, and additionally leave low- and lower-middle income countries as a reservoir for SARS-CoV-2 and possibly emerging variants. Efficient communication strategies are important to increase vaccine confidence and acceptability in the population, who may have a lower perceived risk.

## Supplementary Information

**Additional file 1: Figure S1.** Seropositivity of participants by age-sex stratum. Percentages and numbers in parentheses indicate the proportion of each stratum that is IgG seropositive. Abbreviations: n, nominator of individuals in each stratum; N, denominator of individuals in each stratum; Pos., positive; CI, Confidence Interval.

**Additional file 2: Appendix 1.** Statistical model.

**Additional file 3: Appendix 2.** Stan code.

**Additional file 4: Appendix 3.** SOP Navigation and household identification.

## Data Availability

The datasets generated and analyzed during the current study are not publicly available due to ongoing analyses but are available from the corresponding author on reasonable request. The Statistical Appendix and Stan Code for adjusted seroprevalence estimate was uploaded as supplemental material.
