# Supplementary figures and images for: High seroprevalence of SARS-CoV-2 in Burkina-Faso, Ghana and Madagascar in 2021: a population-based study
Source: BMC Public Health. 2022 Sep 5;22:1676. doi: 10.1186/s12889-022-13918-y (PMC9441841; doi:10.1186/s12889-022-13918-y)

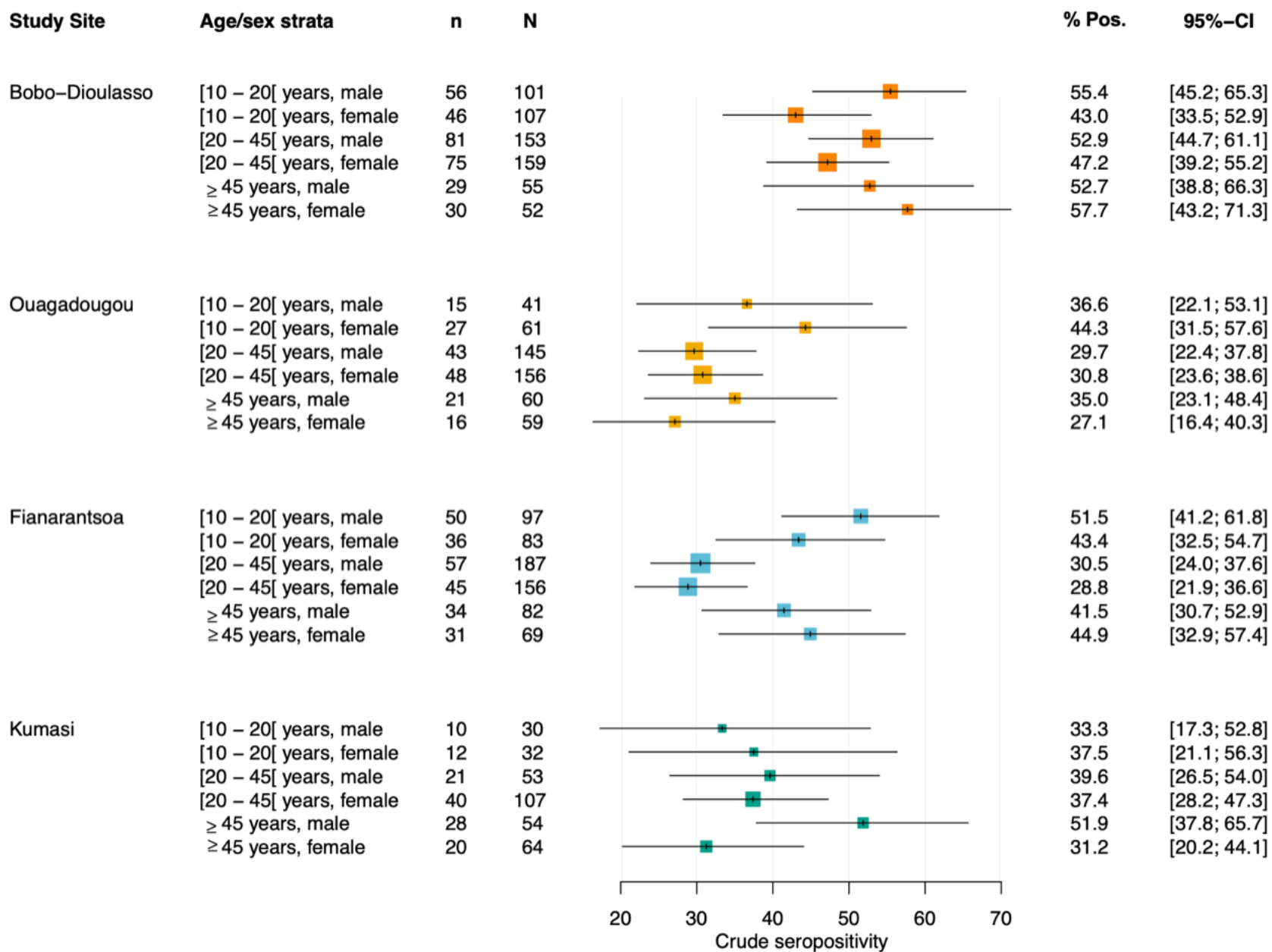

Supplement: Supplementary file 1 — Additional file 1: Figure S1. Seropositivity of participants by age-sex stratum. Percentages and numbers in parentheses indicate the proportion of each stratum that is IgG seropositive. Abbreviations: n, nominator of individuals in each stratum; N, denominator of individuals in each stratum; Pos., positive; CI, Confidence Interval. [file 12889_2022_13918_MOESM1_ESM.pdf]
