# Supplementary material for: High seroprevalence of SARS-CoV-2 in Burkina-Faso, Ghana and Madagascar in 2021: a population-based study
Source: BMC Public Health. 2022 Sep 5;22:1676. doi: 10.1186/s12889-022-13918-y (PMC9441841; doi:10.1186/s12889-022-13918-y)
Supplement: Supplementary file 3 — Additional file 3: Appendix 2. Stan code. [file 12889_2022_13918_MOESM3_ESM.pdf]

Appendix XX. Statistical model

The model estimates prevalence *p*, taking into account specificity and sensitivity of the test and the uncertainty around them:

$$y \sim \text{binomial}(n, p_{sample})$$
$$p_{sample} = p*se+(1-p)(1-sp)$$
$$y_{sp} \sim \text{binomial}(n_{sp},sp)$$
$$y_{se} \sim \text{binomial}(n_{se},se)$$

where p_{sample} is the probability of a positive result, *y* is the number of positives observed, *n* is the sample size and *se* and *sp* are the sensitivity and specificity of the test. Prior information on specificity and sensitivity was taken from the test developer's studies (Table 1). These results were included by adding a hierarchical structure to the model of sensitivity and specificity. Following Gelman and Carpenter, we let the parameter vary according to a hierarchical model where, for any sensitivity study *j* and specificity study *k*, the sensitivity sp_j and specificity sp_k are drawn from a normal distribution on the logistic scale:

$$\text{logit}(se_{kj}) \sim \text{normal}(\mu_{se},\sigma_{se})$$
$$\text{logit}(sp_j) \sim \text{normal}(\mu_{sp},\sigma_{sp})$$

where the hyperparameters $\mu_{sp,}$ σ_{sp}, $\mu_{se,}$ and σ_{se} can be estimated from the data. We fit the model to our data assigning a uniform(0,1) prior to *p*, μ_{sp} and μ_{sp} and weak normal$^+$(0,1) prior to σ_{se} and σ_{sp}.

The full model is a multilevel regression hierarchical model with post-stratification (MRP). Although the data collected was largely representative of the population, MRP allowed us to do minor adjustments to the estimates using census data. The model is based on the above, replacing *p* for p_i(i=1 to n), the result of a logistic regression that models the probability of being positive on age group, subregion and gender:

$$p_i=\text{logit}^{-1}(b_1+b_2*male+a^{age}{}_{age[i]}+a^{subregion}{}_{subregion[i]})$$
$$p_{sample[i]}=p_i*se+(1-p_i)*(1-sp)$$
$$y_i \sim \text{bernoulli}(p_{sample[i]})$$

where male is a variable with the value 1 for male and 0 for female, age[i] and subregion[i] are index variables that correspond to individual i, b_1 and b_2 are logistic regression coefficients and a_{age} and $a_{subregion}$ are vectors of varying intercepts:

$$a_{age} \sim \text{normal}(0, \sigma_{age})$$

$$a_{subregion} \sim \text{normal}(0, \sigma_{subregion})$$

Table 1: Sensitivity and specificity of SARS-CoV-2 IgG ELISAs.

Sample	Months post-onset	Gold standard N	Kit agreement	Details	Origin
Specificity (negative agreement)					
1	n.a.	145	145	1:2 dilution, cut-off 0.40, serum	pre-Covid Ghana, 2014/2015, children
2	n.a.	131	131	1:2 dilution, cut-off 0.40, serum	pre-Covid Ghana, 1999, teens/adults
3	n.a.	167	167	1:2 dilution, cut-off 0.40, plasma	pre-Covid Ghana, 1999, teens/adults
4	n.a.	93	93	concentrated, cut-off 0.36, plasma	pre-Covid Ghana, 1999, teens/adults
5	n.a.	93	93	concentrated, cut-off 0.38	pre-Covid Burkina, 2003/2006/2007, adults
Sensitivity (positive agreement)					
1	<1	19	17	1:2 dilution, cut-off 0.400, serum	PCR positive Germany
2	1-2	21	20	1:2 dilution, cut-off 0.401, serum	PCR positive Germany
3	2-5	34	31	1:2 dilution, cut-off 0.402, serum	PCR positive Germany

Abbreviations: n.a., not applicable; N, sample size.

REFERENCES

Gelman A., Carpenter B. Bayesian analysis of tests with unknown specificity and sensitivity. J R Stat Soc Ser C Appl Stat. 2020; 69: 1269-1283
