# Supplementary material for: High seroprevalence of SARS-CoV-2 in Burkina-Faso, Ghana and Madagascar in 2021: a population-based study
Source: BMC Public Health. 2022 Sep 5;22:1676. doi: 10.1186/s12889-022-13918-y (PMC9441841; doi:10.1186/s12889-022-13918-y)
Supplement: Supplementary file 4 — Additional file 4: Appendix 3. SOP Navigation and household identification. [file 12889_2022_13918_MOESM4_ESM.pdf]

```
// Multilevel regression and poststratification (MRP) to adjust for
// differences between sample and population in terms of age, sex and
// population sizes

data {
  int<lower = 0> N;  // number of tests in the sample
  int<lower = 0, upper = 1> y[N];  // 1 if positive, 0 if negative
  vector<lower = 0, upper = 1>[N] male;  // 0 if female, 1 if male
  int<lower = 1, upper = 4> age[N];  // 1=10-20, 2=20-39, 3=40-59, 4=60+
  int<lower = 0> N_region;  // number of regions within site
  int<lower = 1, upper = N_region> region[N];  // regions within site
  vector[N_region] x_region;  // predictors at the subregion level
  int<lower = 0> J_spec;
  int<lower = 0> y_spec [J_spec];
  int<lower = 0> n_spec [J_spec];
  int<lower = 0> J_sens;
  int<lower = 0> y_sens [J_sens];
  int<lower = 0> n_sens [J_sens];
  int<lower = 0> J;  // number of population cells,
  vector<lower = 0>[J] N_pop;  // population sizes for poststratification
  real<lower = 0> coef_prior_scale;
  real<lower = 0> logit_spec_prior_scale;
  real<lower = 0> logit_sens_prior_scale;
}


parameters {
  real mu_logit_spec;
  real mu_logit_sens;
  real<lower = 0> sigma_logit_spec;
  real<lower = 0> sigma_logit_sens;
  vector<offset = mu_logit_spec, multiplier = sigma_logit_spec>[J_spec]
logit_spec;
  vector<offset = mu_logit_sens, multiplier = sigma_logit_sens>[J_sens]
logit_sens;
  vector[2] b;  // intercept, coef for male, and coef for x_region
  real<lower = 0> sigma_age;
  real<lower = 0> sigma_region;
  vector<multiplier = sigma_age>[4] a_age;  // varying intercepts for age
category
  vector<multiplier = sigma_region>[N_region] a_region;  // varying
intercepts for subregion
}


transformed parameters {
  vector[J_spec] spec = inv_logit(logit_spec);
  vector[J_sens] sens = inv_logit(logit_sens);
}


model {
  vector[N] p = inv_logit(b[1]
                          + b[2] * male
                          + a_age[age]
                          + a_region[region]);
  vector[N] p_sample = p * sens[1] + (1 - p) * (1 - spec[1]);
  y ~ bernoulli(p_sample);
```

```
  y_spec ~ binomial(n_spec, spec);
  y_sens ~ binomial(n_sens, sens);
  logit_spec ~ normal(mu_logit_spec, sigma_logit_spec);
  logit_sens ~ normal(mu_logit_sens, sigma_logit_sens);
  sigma_logit_spec ~ normal(0, logit_spec_prior_scale);
  sigma_logit_sens ~ normal(0, logit_sens_prior_scale);
  mu_logit_spec ~ normal(4, 2);  // weak prior on mean of distribution of
spec
  mu_logit_sens ~ normal(4, 2);  // weak prior on mean of distribution of
sens
  a_age ~ normal(0, sigma_age);
  a_region ~ normal(0, sigma_region);
  // prior on centered intercept
  b[1] + b[2] * mean(male) ~ logistic(0, 1);
  b[2] ~ normal(0, coef_prior_scale);
  sigma_age ~ normal(0, coef_prior_scale);
  sigma_region ~ normal(0, coef_prior_scale);
}


generated quantities {
  real p_avg;
  vector[J] p_pop;  // population prevalence in the J poststratification
cells
  int count;
  count = 1;
  for (i_region in 1:N_region) {
    for (i_age in 1:4) {
        for (i_male in 0:1) {
          p_pop[count] = inv_logit(b[1]
                                   + b[2] * i_male
                                   + a_age[i_age]
                                   + a_region[i_region]);
          count += 1;
        }
    }
  }
  p_avg = sum(N_pop .* p_pop) / sum(N_pop);
}
```
